# Basic research on herpes simplex viruses: are mutants still needed?

**DOI:** 10.1007/s11262-023-02005-y

**Published:** 2023-06-07

**Authors:** Bertfried Matz, Johannes Blümel, Oliver Schildgen, Anna Maria Eis-Hübinger, Hendrik Streeck

**Affiliations:** 1 Institute of Virology, Medical Centre, University of Bonn, Sigmund-Freud-Str. 25, 53127 Bonn, Germany; 2 Paul-Ehrlich-Institut, Paul-Ehrlich-Str. 51-59, 63225 Langen, Germany; 3 Institut für Pathologie, Kliniken der Stadt Köln, Krankenhaus Merheim and Klinikum der Privaten Universität Witten/Herdecke, Ostmerheimer Str. 200, 51109 Cologne, Germany

To the Editor

Herpes viruses are present as blind passengers in the majority of humans and occasionally manifest themselves as insidious foes.

When studying biological processes of herpes simplex viruses in host cells our research group enormously benefitted from a considerable number of virus mutants, that had been expertly isolated/constructed in several notable institutions. Now, we have come to the assessment, that exploiting the scientific potentials of these highly valuable tools would by far exceed our own means.

The collection consists of 49 mutants derived from HSV-1 and HSV-2 standard laboratory strains (Annex I). The affected gene functions have been identified in 28 mutants. The remaining mutants have been partially characterized physically, genetically and/or phenotypically. Forty-one of the 49 mutants have a temperature-sensitive phenotype, ideally suitable for studying dynamic processes in infected cells. They can be propagated at 33 °C, and their genetic lesions are lethal at 39 °C. The parental wild-type strains do grow at both temperatures.

While all mutants were plaque-purified we strongly recommend to regularly check for mycoplasma when preparing own stocks, as this was not regularly done for all original stocks. While the phenotypes of all strains are characterized and the majority of strains has literature reference, no full length sequences have been published or deposited, yet.

Informations on the isolation history (scientist/institution), genetic defect, and prior use in research projects may be found in the Annex I to this letter. Virus samples may be provided by our Institute under the conditions that a mutant (i) must not be renamed and (ii) its origin must be named (i.e. investigator and institution), when used in a project/publication. It is also foreseen to deposit the variants at the UK culture collection virus biobank (www.culturecollections.org.uk) for non-commercial usage.

## Annex I

### Herpes simplex virus: a collection of some mutants

#### Preface

Our investigations on herpesviral interactions with host cells have been enabled by virus mutants we had gratefully received from several renowned virologists (see list below). Having realized the high value of temperature-sensitive (ts) virus mutants for many kinds of research projects, we are carefully keeping them stored for a potential use by any scientist in future. They may be provided by the Institute of Virology at Bonn under the condition that the mutant (i) must not be renamed, and the institution with the investigator who isolated it (ii) must be mentioned if published. The references (numbers in brackets) are but a tiny selection of published data on the mutants in our collection. Further specific or general information, e.g. on handling of mutants, may be requested from us (see address for correspondence).

##### Derivatives of parental strain HSV-1 17+

**tsA**/ts lesion in **glycoprotein B (UL27)**/isolated by: J. H. Subak-Sharpe, Glasgow/ts lesion identified [1]/ protein data [2]/used in research projects: complementation analyses [3–5]; initiation and mode of herpesviral DNA replication [6]; HSV-induced amplification of heterologous (i.e. non-herpesviral) DNA [7, 8].

**tsD**/ts lesion in **major transactivating protein (ICP4)**/isolated by J. H. Subak-Sharpe, Glasgow/ts lesion identified [1]/protein data [2]/used in research projects: Complementation analyses [3, 5]; Host cell stress protein activation [9, 10].

**tsG**/**DNA + **/isolated by J. H. Subak-Sharpe, Glasgow/protein data [2]/ used in research projects: Complementation analyses [3, 5].

**tsH**/ts lesion in **DNA polymerase (UL30)**/isolated by: I. Crombie, Glasgow/ts lesion identified [11]/protein data [2]/used in research projects: complementation analyses [4] Initiation and mode of herpesviral DNA replication [6, 12, 13]; HSV-induced amplification of heterologous (i.e. non-herpesviral) DNA [7, 8, 14–16].

**tsK**/ts lesion in **major transactivating protein (ICP4)**/isolated by I. Crombie, Glasgow/ts lesion identified [1, 17]/protein data [2]/used in research projects: Transcriptional regulation of herpesviral genes [18]; Host cell stress protein activation [10]; HSV-induced amplification of heterologous (i.e. non-herpesviral) DNA [7, 8, 16]; HSV-induced morphological transformation of host cells [19].

**tsO**/ts lesion in **99 kD component (UL5) of the heterotrimeric helicase-primase complex**/isolated by R. Thompson, D. Dargan and J. H. Subak-Sharpe, Glasgow/ts lesion identified [4]/protein data [2]/used in research projects: Complementation analyses [4]; Requirement of HSV genes essential for viral DNA synthesis [20]; Interaction of herpesviral and polyomaviral DNA replication factors [21]; Initiation and mode of herpesviral DNA replication [6, 12].

**tsR**/ts lesion in **origin-binding protein (UL9)**/isolated by R. Thompson, D. Dargan and J. H. Subak-Sharpe, Glasgow/ts lesion identified [4, 13]/protein data [2, 22, 23]/used in research projects: Complementation analyses [4]; Requirement of HSV genes essential for viral DNA synthesis [20]; Initiation and mode of herpesviral DNA replication [6, 12, 13, 23]; HSV-induced morphological transformation of host cells [24].

**tsS**/ts lesion in **origin-binding protein (UL9)**/isolated by I. Crombie, Glasgow /ts lesion identified [1, 4, 13]/protein data [2, 22, 23]/used in research projects: Complementation analyses [4]; Initiation and mode of herpesviral DNA replication [6, 12, 13, 23, 25]; HSV-induced morphological transformation of host cells [24]; HSV-induced amplification of heterologous (i.e. non-herpesviral) DNA [8, 14].

**tsX**/ts lesion in **origin-binding protein (UL9)**/isolated by J. H. Subak-Sharpe, Glasgow/ts lesion identified [4, 13]/protein data [2]/used in research projects: Initiation and mode of herpesviral DNA replication [12, 13]; HSV-induced morphological transformation of host cells [19, 24].

**tsVP1206**/ts lesion in **114kD component (UL52) of the heterotrimeric helicase-primase complex**/isolated by V. G. Preston, Glasgow/ ts lesion identified [4]/used in research projects: Complementation analyses [4]; Requirement of HSV genes essential for viral DNA synthesis [20]; Initiation and mode of herpesviral DNA replication [12].

**IP2**/phosphonoacetic acid (PAA) resistant mutation in **DNA polymerase (UL30**/Isolated by NN, Glasgow/mutation mapped [11]/used in research projects: HSV-induced amplification of heterologous (i.e. non-herpesviral) DNA [7, 14].

**HSV-1 in1411**/lethal mutation in the **major transactivating protein ICP4**/constructed by N. D. Stow, Glasgow: termination codon inserted at position 82 > truncated polypeptide; propagation of virus requires complementing cells expressing ICP4/used in research project: Host cell stress protein activation [26]; HSV-induced morphological transformation of host cells [19].

##### Derivatives of parental strain HSV-1 KOS

**ts164** (N20) Complementation Group 1–13/ts lesion in **a DNA packaging function (UL32)**/Isolated by P. A. Schaffer, Boston/ts lesion 0.443–0.448 [27]/used in research project: viral and cellular DNA synthesis [28]; virus maturation process [29].

**ts343** (A1) Complementation Group 1–1/ts lesion in **major DNA-binding protein (UL29)**/Isolated by P. A. Schaffer, Boston/ used in research project: viral and host cell DNA synthesis [28]; complementation study and replication analysis [30] HSV-induced amplification of heterologous (i.e. non-herpesviral) DNA [15].

**ts372** (Q26) Complementation Group 1–24/**DNA ± **/Isolated by P. A. Schaffer, Boston/ts lesion 0.301–0.304 [31]/used in research project: Complementation analyses [31].

**ts480** (A16) Complementation Group 1–1/ts lesion in **major DNA-binding protein (UL29)**/Isolated by P. A. Schaffer, Boston/used in research project: viral and host cell DNA synthesis [28]; complementation study and replication analysis [30]; HSV-induced amplification of heterologous (i.e. non-herpesviral) DNA [12, 15, 16].

**ts501** (F17) Complementation Group 1–6/**DNA + **/ Isolated by P. A. Schaffer, Boston/ts lesion 0.086–0.103 [32]/used in research project: viral and host cell DNA synthesis [28].

**ts847** (B2) Complementation Group 1–2/ts lesion in **major transactivating protein (ICP4)**/Isolated by P. A. Schaffer, Boston/ used in research project: viral and host cell DNA synthesis [28].

**ts867** (C4) Complementation Group 1–3/ts lesion in **DNA polymerase (UL30)** /Isolated by P. A. Schaffer, Boston/used in research project: viral and host cell DNA synthesis [28] HSV-induced amplification of heterologous (i.e. non-herpesviral) DNA [15].

**ts953** (V37) Complementation Group 1–29/**DNA + **/Isolated by P. A. Schaffer, Boston/ts lesion 0.53–0.594 [33].

**ts1112** (E6) Complementation Group 1–5/ts lesion in **regulatory protein ICP27 (UL54)**/Isolated by P. A. Schaffer, Boston//used in research project: viral and host cell DNA synthesis [28]; Regulation of herpesviral gene expression [34].

**ts1136** (C7) Complementation Group 1–3/ts lesion in **DNA polymerase (UL30)**/Isolated by P. A. Schaffer, Boston/used in research project: viral and host cell DNA synthesis [28]; HSV-induced amplification of heterologous (i.e. non-herpesviral) DNA [15].

**ts1178** (G8) Complementation Group 1–7/ts lesion in **major capsid protein VP5 (UL19)**/Isolated by P. A. Schaffer, Boston/ts lesion 0.103–0.186 [27].**ts1585** (D9) Complementation Group 1–4/ts lesion in **DNA polymerase (UL30)**/Isolated by Priscilla Schaffer, Boston/used in research project: Complementation analyses [5].

**ts1789** (I11) Complementation Group 1–8/**DNA + **/Isolated by P. A. Schaffer, Boston/used in research project: Complementation analyses [5].**hr27** Complementation Group 1–36/lethal defect in **origin-binding protein (UL9)**/host range mutant constructed by S. K. Weller, Farmington/propagation of virus requires complementing cells expressing UL9/used in research project: herpesviral DNA replication [35]; HSV-induced amplification of heterologous (i.e. non-herpesviral) DNA [36].

**hr80** Complementation Group 1–26/lethal defect in **80 kD component (UL8) of the heterotrimeric helicase-primase complex**/host range mutant constructed by S. K. Weller, Farmington/propagation of virus requires complementing cells expressing UL8/used in research project: herpesviral DNA replication [35]; HSV-induced amplification of heterologous (i.e. non-herpesviral) DNA [36].

**hr114** Complementation Group 1–37/lethal defect in **114 kD component (UL52) of the heterotrimeric helicase-primase complex**/host range mutant constructed by S. K. Weller, Farmington/propagation of virus requires complementing cells expressing UL52/used in research project: herpesviral DNA replication [35]; HSV-induced amplification of heterologous (i.e. non-herpesviral) DNA [36].

**PAA**^**r**^**5**/phosphonoacetic acid (PAA) resistant mutation in **DNA polymerase (UL30)**/isolated by D. M. Coen, Boston.

**KOSacg**^**r**^/acycloguanosine (ACG) resistant mutation /isolated by D. M. Coen, Boston.

##### Derivatives of parental strain HSV-1 HFEM

**tsLB1**/**DNA + **/isolated by I. W. Halliburton, Leeds/used in research project: Complementation analyses [5].

**tsLB2**/**DNA ± **/isolated by I. W. Halliburton, Leeds/used in research project: Complementation analyses [5].

**tsLB3**/**DNA + **/isolated by I. W. Halliburton, Leeds/used in research project: Complementation analyses [5].

**tsLB4**/**DNA + **/ isolated by I. W. Halliburton, Leeds / used in research project: Complementation analyses [5].

**tsLB5**/**DNA + **/isolated by I. W. Halliburton, Leeds/used in research project: Complementation analyses [5].

**tsLB7**/**DNA?**/isolated by I. W. Halliburton, Leeds/.

**tsLS1**/**DNA + **/ isolated by I. W. Halliburton, Leeds / used in research project: Complementation analyses [5].

**tsLS2**/**DNA + **/ isolated by I. W. Halliburton, Leeds / used in research project: Complementation analyses [5].

##### Derivative of parental strain HSV-1 A44

**ts2A44**/ts lesion in **capsid assembly protein VP19c (UL38)**/isolated by P. Sheldrick, Villejuif/used in research project: herpesviral particle maturation, DNA packaging [37, 38].

##### Derivative of parental strain HSV-1 ANGpath

**mut12**/84 bp deletion in **gene encoding the major transactivating protein ICP4**/Isolated by C. H. Schröder, Heidelberg/used in research project: regulation of herpesviral gene expression [39].

##### Derivatives of parental strain HSV-2 HG52

**ts1**/**DNA − **/Isolated by M. Timbury, Glasgow/ts lesion ca. 0.62–0.72 [40]/used in research project: Complementation analyses [5]; Enzymes in herpesvirus-infected cells [41]; Virus pathogenicity and latency in prosimians [42].

**ts2**/**DNA − **//Isolated by M. Timbury, Glasgow/ used in research project: Complementation analyses [5]; Enzymes in herpesvirus-infected cells [41]; Virus pathogenicity and latency in prosimians [42].

**ts5**/**DNA + **/Isolated by M. Timbury, Glasgow/ts lesion ca. 0.26–0.38 [40]/used in research project: Complementation analyses [5]; Enzymes in herpesvirus-infected cells [41]; Virus pathogenicity and latency in prosimians [42].

**ts6**/**DNA − **/isolated by M. Timbury, Glasgow/ts lesion ca. 0.4–0.42 (7/used in research project: Enzymes in herpesvirus-infected cells [41].

**ts9**/**DNA − **/Isolated by M. Timbury, Glasgow/ts lesion ca. 0.44–0.53 [40]; /used in research project: Complementation analyses [5]; Enzymes in herpesvirus-infected cells [41]; Virus pathogenicity and latency in prosimians [42].

**ts10**/**DNA − **/ Isolated by M. Timbury, Glasgow/used in research project: Enzymes in herpesvirus-infected cells [41]; Virus pathogenicity and latency in prosimians [42].

**ts12**/**DNA ± **/Isolated by M. Timbury, Glasgow/used in research project: Complementation analyses [5]; Enzymes in herpesvirus-infected cells [41]; Virus pathogenicity and latency in prosimians [42].

**ts13**/**DNA ± **/Isolated by M. Timbury, Glasgow/ts lesion ca. 0.65–0.7 [40]/used in research project: Complementation analyses [5]; Enzymes in herpesvirus-infected cells [41]; Virus pathogenicity and latency in prosimians [42].

##### Derivative of parental strain HSV-2 186

**tsH9**/ts lesion in major** DNA-binding protein**/isolated by D. J. M. Purifoy/used in research project: Complementation analyses [5].

## Declarations

### Scientists who isolated/constructed the HSV mutants that were generously provided to our research group

**Coen, Donald M.:** Department of Pharmacology, Harvard Medical School, Boston MA (USA).

**Crombie, Iain K.:** Medical Research Council, Virology Unit, Institute of Virology, University of Glasgow (UK).

**Dargan, Derek:** Medical Research Council, Virology Unit, Institute of Virology, University of Glasgow (UK).

**Halliburton, Ian W.:** Department of Microbiology (Virology), University of Leeds (UK).

**Preston, Valerie G.:** Medical Research Council, Virology Unit, Institute of Virology, University of Glasgow (UK).

**Purifoy, Dorothy J. M.:** Department of Microbiology (Virology) University of Leeds (UK).

**Schaffer, Priscilla A.:** Sidney Farber Cancer Institute, Dana Farber Cancer Institute, Department of Microbiology and Molecular Genetics, Harvard Medical School, Boston MA (USA).

**Schröder, Claus H.:** Institute of Virus Research, German Cancer Research Center, Heidelberg (Germany).

**Sheldrick, Peter:** Institut de Recherches Scientifiques sur le Cancer, Villejuif (France).

**Stow, Nigel D.:** Medical Research Council, Virology Unit, Institute of Virology, University of Glasgow (UK).

**Subak-Sharpe, John H.:** Medical Research Council, Virology Unit, Institute of Virology, University of Glasgow (UK).

**Thompson, Russell:** Medical Research Council, Virology Unit, Institute of Virology, University of Glasgow (UK).

**Timbury, Morag C.:** Medical Research Council, Virology Unit, Institute of Virology, University of Glasgow (UK).

**Weller, Sandra K.:** Department of Microbiology, University of Connecticut Health Center, Farmington CT (USA).
